# Reproductive Characteristics of *Odontobutis potamophila*: Implications for Sustainable Fisheries Management

**DOI:** 10.3390/ani15213150

**Published:** 2025-10-30

**Authors:** Miao Xiang, Shasha Zhao, Bo Li, Li Li, Man Wang, Jie Wang, Ruru Lin, Lei Zhang

**Affiliations:** 1 Yangtze River Fisheries Research Institute, Chinese Academy of Fishery Sciences, Wuhan 430223, China; xiangmiaoihb@163.com; 2 Shandong Engineering Research Center of Green and High-Value Marine Fine Chemical, School of Chemical Engineering and Environment, Weifang University of Science and Technology, Weifang 262700, China; zhaoshasha1131@126.com; 3 Fisheries Research Institute, Wuhan Academy of Agricultural Sciences, Wuhan 430207, China; xiaowu18827408689@163.com; 4 Institute of Hydrobiology, Chinese Academy of Sciences, Wuhan 430072, China; 15189658980@163.com (L.L.); wangman1@ihb.ac.cn (M.W.); wangjie@ihb.ac.cn (J.W.); linruru23@mails.ucas.ac.cn (R.L.); 5 Huai’an Research Centre, Centre of Hydrobiology, Huai’an 223002, China

**Keywords:** fecundity, *Odontobutis potamophila*, male care, reproductive traits

## Abstract

**Simple Summary:**

A one-year study of *Odontobutis potamophila* in Nansi Lake found that this species matures at one year and around 73.6 mm, spawns primarily from March to June (peaking in May), and depends on individuals aged two years and older for more than 80% of total egg production. To promote sustainable populations, we recommend implementing a seasonal fishing closure from March to June, establishing a minimum catch size of 80 mm, and utilizing peak GSI values in May to optimize hatchery operations. These measures aid in conserving this ecologically significant benthic fish during crucial reproductive phases.

**Abstract:**

*Odontobutis potamophila*, a small benthic carnivorous fish endemic to the Yangtze River basin, holds considerable ecological and commercial value. However, overfishing and habitat degradation have led to a severe decline in its wild population. A lack of quantitative reproductive data has further hampered effective conservation and resource management. To address this, we conducted monthly sampling, collecting a total of 894 individuals from Nansi Lake between August 2017 and July 2018. By integrating gonadal histological staging, gonadosomatic index (GSI) analysis, logistic regression, and fecundity assessments, we provide a foundational understanding of the species’ reproductive biology. The annual sex ratio was 1.06:1, with a temporary female bias in April (2.14:1) shifting due to male nest-guarding behavior. Both sexes reached maturity at one year and approximately 73.6 mm in length. Spawning occurred from March to June, peaking in May (GSI = 28.92%). Absolute fecundity ranged 2306 ± 1430 eggs and correlated positively with body size and age, while relative fecundity stabilized after age two. Individuals aged two years and older contributed over 80% of total egg production, reflecting a strategy of early maturation with high reproductive output at older ages. This study aims to systematically understand the reproductive biology of *O. potamophila*. These results support science-based measures such as Covering the entire window from gonadal maturation to fry dispersal, an annual fish ban established from March to June, a minimum catch size of 80 mm, and improved broodstock management for aquaculture and conservation efforts aimed at this and related benthic fishes in shallow lake ecosystems.

## 1. Introduction

Reproductive biology is fundamental to understanding fish life history, linking individual physiology with population level strategies. It plays a critical role in determining a population’s resilience and regenerative capacity under environmental stress [1,2]. Imbalances in sex ratios, impaired gonadal development, delayed maturation, and reduced reproductive output can interact with density-dependent mechanisms, potentially leading to population instability or collapse [3,4,5,6,7,8]. External factors such as temperature, nutrition, hydrology, and fishing pressure regulate these traits through neuroendocrine pathways, energy allocation trade-offs, and behavioral adaptations [6,9,10,11,12,13]. For economically significant species, reproductive parameters directly influence artificial breeding outcomes, including induction, fertilization, and seedling survival rates, as well as the economic efficiency and genetic gains in selective breeding programs [14,15,16]. Insufficient reproductive data often perpetuates a cycle of overfishing, population decline, and unsuccessful restocking, exacerbated by suboptimal practices in spawning timing, sex ratios, or nutrition [17,18]. Genetic diversity alone cannot break this cycle; only by filling the gaps in reproductive biology data can precise conservation strategies be developed. Thus, detailed investigation of species-specific reproductive traits is crucial for deciphering adaptive responses to environmental change and for scientifically guiding population management, conservation efforts, and aquaculture development.

Nansi Lake, a key storage lake in Shandong Province for the South-to-North Water Diversion Project’s East Route, spans approximately 1266 km^2^ and represents a typical shallow lake ecosystem in northern China [19,20]. The lake’s pronounced annual water-level variations promote the development of diverse habitats, including shallow shoals and wetlands. These structurally complex microenvironments support abundant benthic, planktonic, and macrophyte communities, which in turn sustain a rich fish assemblage and contribute to stable fishery yields [21,22].

The dark sleeper, *Odontobutis potamophila* is widely distributed in the slow-flowing or shallow waters of the eastern Chinese freshwater systems [23,24]. This benthic ambush predator feeds mainly on crustaceans, small fish, and aquatic insects. It demonstrates strong physiological adaptations, such as broad temperature tolerance, resilience to low-oxygen conditions, and rapid growth, contributing to its high economic value and market appeal [24,25,26,27,28,29,30]. These characteristics make it a promising species for aquaculture development [25,26]. Although previous studies have focused on genetic diversity, chromosome karyotypes, and early developmental morphology in certain regional populations [24,27,28,29,30], the reproductive biology of this species, especially in shallow lake habitats, remains poorly understood [23,25]. Key reproductive parameters such as sex ratio, annual gonadal development, size at first maturity, and fecundity have not been quantitatively studied. The absence of these critical data hinders the optimization of artificial breeding, particularly in determining induced-spawning timing and broodstock ratios, thereby limiting large-scale seed production and conservation efforts.

Based on year-round monthly surveys in Nansi Lake, this study integrated histological staging, gonadosomatic index dynamics, and logistic maturation modeling to systematically quantify sex ratio, gonadal development, size at first maturity, and reproductive capacity of *O. potamophila*. The results confirm a shallow-water adaptation strategy characterized by small body size, early maturation, and high reproductive investment, elucidating the species’ reproductive tactics and energy allocation mechanisms in shallow lake ecosystems. The findings provide directly applicable biological benchmarks for establishing fish ban periods, setting harvestable size limits, and implementing broodstock management.

## 2. Materials and Methods

### 2.1. Study Area

**Figure 1:**
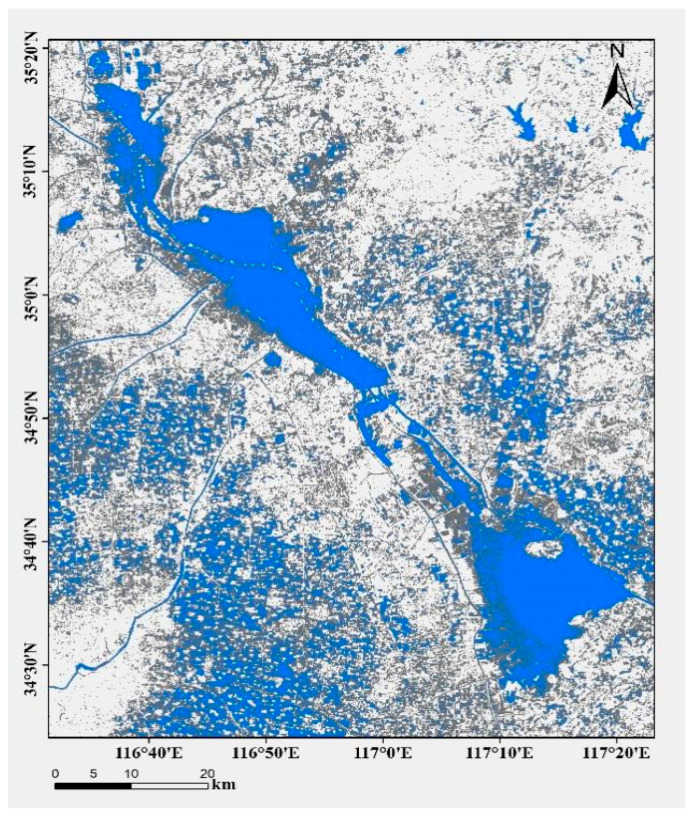
Study area—Nansi Lake.

Nansi Lake (116°34′–117°21′ E, 34°27′–35°20′ N) consists of four interconnected sub-lakes: Nanyang, Dushan, Zhaoyang, and Weishan. The lake has a mean depth of approximately 1.5 m, though areas south of Weishan Island reach around 3 m. Oriented north–south, the lake is divided by a dam structure into two main sections: the upper lake, which is 67 km long with an area of 602 km^2^, and the lower lake, extending 58 km in length and covering 664 km^2^ (Figure 1). Sampling was conducted monthly at fixed stations in Nansi Lake Han Zhuang Waters from August 2017 to July 2018, with the exception of February 2018 due to ice cover. All sampling was conducted in situ on the same day without any artificial heating or cooling of the water bodies. Environmental indicators were also maintained in their natural state, accurately reflecting the regulatory effects of natural temperature and environment on gonadal maturation. The fish were captured using benthic trap nets, each measuring 15 m in length and 0.6 m in width with a mesh size of 4 mm. The nets were set in the evening and retrieved after 12 h [31]. The samples were transported on ice to the laboratory for further processing and analysis.

### 2.2. Sample Processing

Each month, the standard length (SL, 0.1 mm) and body weight (BW, 0.01 g) of all collected individuals were measured. Age was determined via otolith analysis, with the study focusing exclusively on reproductively active individuals [23]. Sex was initially identified based on the genital pore, followed by comprehensive dissection and macroscopic examination of the gonads. Developmental stages of the gonads were classified according to external features including color, size, yolk deposition, and degree of spermatid filling [32,33] (Table 1). Sex was determined by the presence of gonads at stage II or later. Supplementary criteria included abdominal protrusion and genital pore morphology during the breeding season. Morphological staging revealed that early-stage gonads are minute; they gradually enlarge as development proceeds. At maturity, ovaries occupy most of the body cavity with eggs visible to the naked eye, whereas testes appear as yellowish-white, densely vascularised bands. After ovulation or spermiation, the gonads rapidly shrink and become flaccid, showing a marked reduction in volume [3,34,35]. The gonads (gonad weight, W_G_) were weighed, and the empty weight (net weight, W_N_) was measured after the removal of internal organs and gonads to compute the gonadosomatic index (GSI) [36]. Since we use eviscerated weight for calculations, the GSI values obtained here will be elevated:

$$\mathrm{GSI}\;=\;\frac{W_{G}}{W_{N}}\;\times \;100\%$$

Based on the length-age data from January to June, each 10 mm of body length was grouped together, and the *SL*_50%_ method was applied to calculate the maturity ratio of each group [37]. The initial sexual maturity length of male and female individuals was determined by fitting the Logistic curve equation:

$$P_{i}\;=\;1/\left(1\;+\;e^{-m\left(\mathrm{SL}-{\mathrm{SL}}_{50\%}\right)}\right)$$

In the formula: P_i_ is the body length group, *SL_i_* is the percentage of gonadal maturity; *m* is the maturity growth coefficient (The estimated values obtained by fitting the logistic curve equation were 0.09 for males and 0.03 for females); *SL*_50%_ is the body length at 50% sexual maturity [38].

Ovaries from mature females (gonadal stages IV and V) were used to estimate absolute fecundity and relative fecundity. After confirming that the left and right ovaries were similar in size, approximately 0.3 g of oocytes were sub-sampled from the anterior, middle, and posterior sections of the right ovary. The total number of oocytes (n) in the sub-sample was counted under a dissecting microscope with visible light inspection (Olympus, SZX16, manufactured by Olympus Corporation, Tokyo, Japan, 2× magnification). The oocytes were then thoroughly mixed, weighed (oocyte weight, Wo), and preserved in 5% formaldehyde. Absolute fecundity (AF) was calculated using the following formula [36]:

$$\mathrm{AF}\;=\;n\;\times \;\frac{W_{G}}{W_{o}}$$

In the formula: n is the number of eggs in sub-sample.

Relative fecundity (RF) was derived as

$$\mathrm{RF}=\frac{\mathrm{AF}}{\mathrm{BW}}$$

**Table 1:** Macroscopic characteristics of gonadal developmental stages of *Odontobutis potamophila*.

Stages	Female	Male
I	Semi-transparent ribbon-like, extremely small, closely attached to both sides of the abdominal membrane on the back, male and female cannot be distinguished.	
II	Strip-shaped, slightly enlarged compared to stage I, but with no significant changes.	Linear or thin ribbon-like, more enlarged than stage I, with inconspicuous blood vessels.
III	Sac-shaped, with small white particles visible inside, slightly yellowish in color.	Strip-like, pale yellow, with obvious blood vessels.
IV	Oocytes clearly visible, yellowish-white in color, with thick, white ovarian walls.	Flat strip-like, with almost equal width at both ends, slightly yellowish.
V	Ovarian walls very thin and soft, with evidently enlarged oocytes, yellowish-white in color. Light pressure on the abdomen can release free oocytes [39].	Flat ribbon-like, with very obvious crisscrossing blood vessels, Upon slight pressure applied to the anterior cloacal region, milky-white semen can be expelled [40].
VI	The ovaries show reduced volume and softened tissue, with visible capillary congestion.	The testicles appear flaccid, with a noticeable reduction in volume.

### 2.3. Data Processing and Analysis

The χ^2^-test was employed to assess whether the observed sex ratio of *O. potamophila* deviated significantly from the theoretical 1:1 ratio. One-way ANOVA was applied to examine differences in body length and weight between sexes, as well as monthly variations in the gonadosomatic index (GSI) of females. The relationships between absolute fecundity and both body length and weight were analyzed using linear regression. Data are expressed as mean ± standard deviation (mean ± SD). All datasets were tested for normality, and non-parametric alternatives were employed when heterogeneous variance was observed. However, since the raw data for body weight in both genders did not satisfy the assumption of normal distribution, and remained non-normally distributed even after log transformation, nonparametric tests were selected. Analyses and graphing were performed with SPSS 19.0 and Origin Pro 8.0, respectively, with significance set at *p* < 0.05.

## 3. Results

### 3.1. Sex Ratio, Body Length, and Body Weight

A total of 894 *O. potamophila* specimens were collected, comprising 433 females, 408 males, and 53 individuals whose sex could not be determined, yielding a female:male ratio of 1.06:1. The χ^2^-test indicated no significant deviation from the expected 1:1 ratio (*p* > 0.05). However, monthly analysis revealed a significant higher proportion of females in April (sex ratio = 2.14, *p* < 0.05), while no significant differences were observed in other months (Table 2).

**Figure 2:**
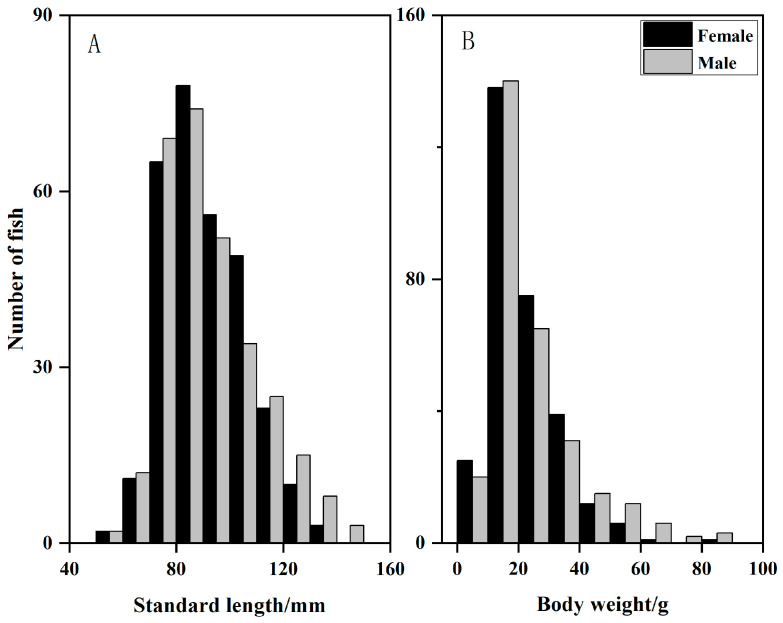
Frequency distributions of standard length (**A**) and body weight (**B**) for *Odontobutis potamophila* sampled from Nansi Lake.

A total of 644 individuals were dissected, consisting 294 males, 297 females, and 53 individuals of undetermined sex. Several fish of unidentified sex were primarily juveniles; since their gonads were in stage II or earlier at the time of dissection, sex could not be determined. Specimens with body lengths of 80–90 mm and body weights of 10–20 g were most abundant, while as larger individuals were relatively uncommon (Figure 2). Owing to significant departures from normality (Kolmogorov–Smirnov test, *p* < 0.05), sexual differences in body size were evaluated with a Mann–Whitney U test. Males averaged 91.03 ± 14.97 mm in length and 23.72 ± 15.60 g in mass, whereas females averaged 92.21 ± 17.73 mm and 21.51 ± 11.28 g, respectively; neither length (*p* = 0.926) nor weight (*p* = 0.316) differed significantly between sexes. To rigorously examine sexual dimorphism, comparisons should be made between male and female individuals within the same age group. Detailed comparisons can be conducted after obtaining age data through otolith identification [23].

### 3.2. Sexual Development and Breeding Season

**Figure 3:**
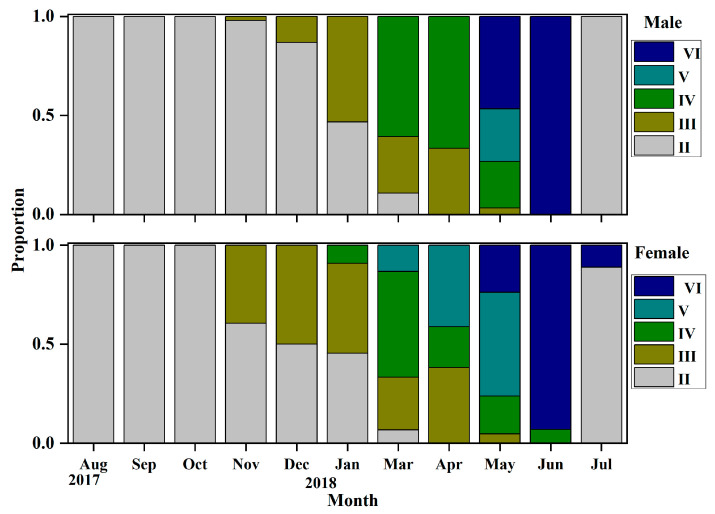
Monthly gonadal maturity stages of male and female *Odontobutis potamophila* in Nansi Lake (August 2017—July 2018).

**Figure 4:**
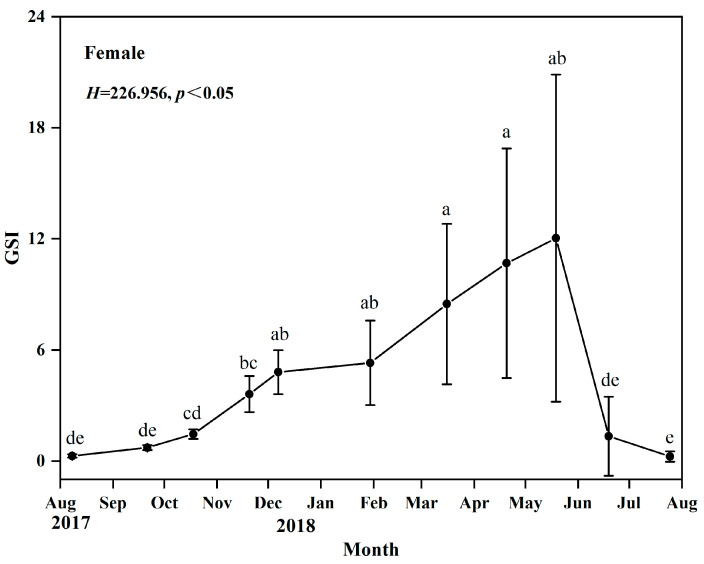
Monthly variation in the gonadosomatic index (GSI) of female *Odontobutis potamophila* in Nansi Lake (August 2017—July 2018) (different letters mean that there existed a significant difference).

Through visual observation and based on their gonadal development characteristics, it can be determined that the gonadal development of *O*. *potamophila* exhibits seasonal fluctuations, with females serving as representative examples. From August to September, ovaries are predominantly in stage II; from October to December, stage III gradually increases; and by January of the following year, stage IV gonads are observed. Between March and June, stages IV, V, and VI constitute the majority, while a small proportion of ovaries remain at stage VI as late as July. Males display a similar developmental pattern, indicating synchronous reproductive cyclical between sexes (Figure 3). Concurrently, annual monitoring of the female gonadosomatic index (GSI) revealed values ranging from 0.03% to 28.92%. The GSI increased gradually from August to December, followed by a rapid linear rise beginning in March, peaking in May, and declining sharply to its lowest point after spawning in June and July. These findings indicate that the breeding season of *O. potamophila* in Nansi Lake spans from March to June, with reproductive activity reaching its peak in May (Figure 4).

### 3.3. Initial Sexual Maturity Body Length

**Figure 5:**
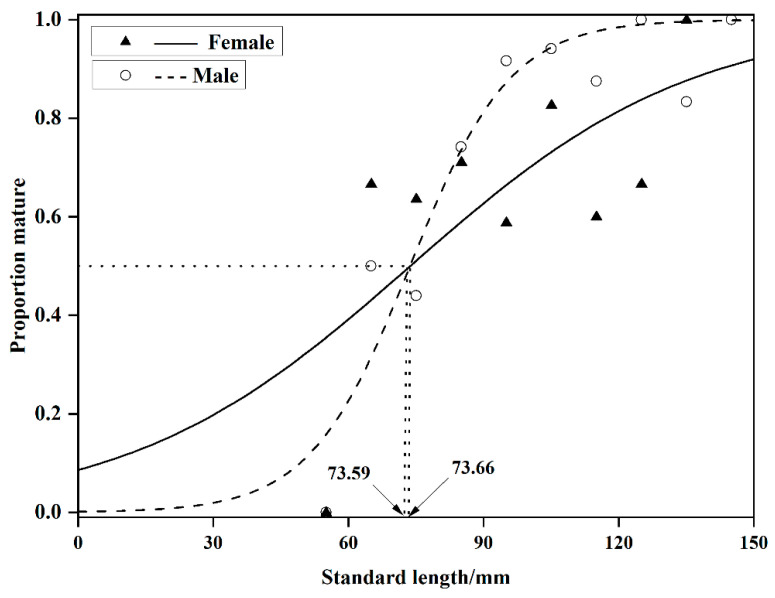
Proportion of mature *Odontobutis potamophila* by 10 mm size intervals in Nansi Lake (August 2017—July 2018).

Based on the relationship between the sex ratio of sexually mature individuals and body length in *O. potamophila*, Logistic curve equations were fitted to estimate the initial sexual maturity body lengths. For females, the equation was $P\;=\;1/\left(1\;+\;e^{-0.03\left(\mathrm{SL}-73.59\right)}\right)$(*R*^2^ = 0.47, n = 136) and for males, $P\;=\;1/\left(1\;+\;e^{-0.09\left(\mathrm{SL}-73.66\right)}\right)$(*R*^2^ = 0.89, n = 126). The initial sexual maturity body lengths were determined to be 73.59 mm for females and 73.66 mm for males (Figure 5).

### 3.4. Fecundity

A total of 59 individuals were used to assess reproductive capacity, with body lengths ranging from 66.08 to 135.96 mm and weights from 8.40 to 85.32 g. The absolute fecundity (AF) of *O. potamophila* ranged from 643 to 8904 eggs, with a mean (±SD) of 2306 ± 1430 eggs. Relative fecundity (RF) varied from 31.84 to 186.36 eggs/g, averaging 91.20 ± 40.09 eggs/g. Analysis across age groups showed that 1-year-old individuals had a mean AF of 1819 ± 839 eggs and a mean RF of 94.87 ± 43.10 eggs/g. Among 2-year-old individuals, mean AF was 3176 ± 1319 eggs and mean RF was 81.89 ± 31.92 eggs/g. Three-year-old individuals exhibited a mean AF of 6006 ± 4097 eggs and a mean RF of 84.07 ± 28.69 eggs/g.

The relationships between absolute fecundity (AF) and body length (*SL*) or body weight (BW) were described by the following equations:

$$AF=1.29{\mathrm{SL}}^{2}-193.16SL+8692.4\;(R^{2}\;=\;0.50,\;n=\;59)$$

$$AF=0.82{\mathrm{BW}}^{2}+14.58BW+1170.5\;(R^{2}\;=\;0.61,\;n=\;59)$$

**Figure 6:**
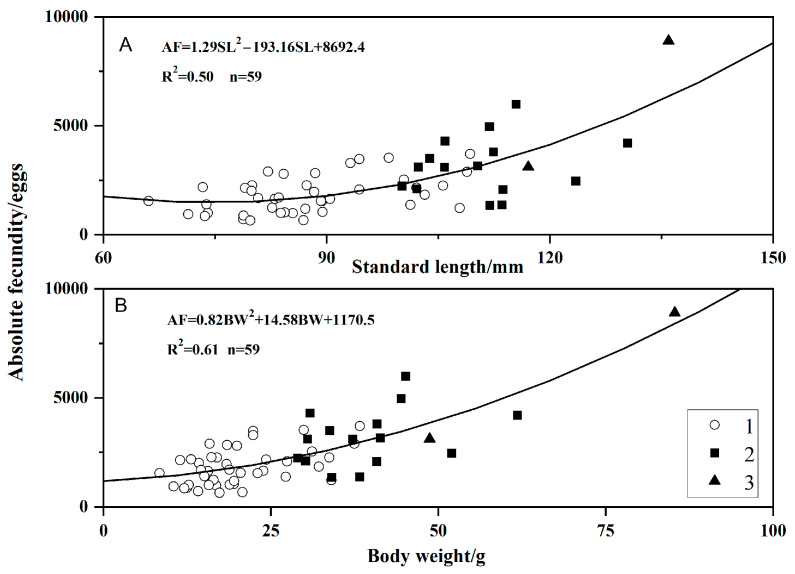
Relationships of absolute fecundity to standard length (**A**) and body weight (**B**) by age class in *Odontobutis potamophila* from Nansi Lake (August 2017–July 2018) (open circle, age 1; black squares, age 2; black triangles, age 3).

Absolute fecundity increased with standard length and body weight, showing progressively greater gains from age-1 to age-3 (Figure 6).

## 4. Discussion

### 4.1. Sex Ratio and Gonadal Coordination Pattern: Reproductive Characteristics Driven by Male Nest-Guarding Behavior

The annual sex ratio of *O. potamophila* in Nansi Lake did not deviate significantly from 1:1 (χ^2^ = 0.372, *p* > 0.05), which is consistent with the typical pattern observed in successfully reproducing populations of bony fishes [41,42]. However, notable temporal fluctuations occurred during the breeding season. In April, the female:male ratio was significantly elevated (F:M = 2.14, *p* < 0.05), while in May, the ratio shifted in favor of males (F:M = 0.74). Such variations in sex ratio may arise from multiple factors including differential growth rates, mortality, longevity, seasonal influences, and potential sex reversal [43,44]. In this population, the transient skew is likely linked to male reproductive behavior, specifically nest guarding [45]. By mid-to-late March, males establish nests within vacant clam shells or rock crevices. Females are attracted to these nests through visual and chemical cues, deposit their eggs, and then quickly depart to participate in additional spawning events [45,46]. In contrast, males remain to guard the eggs. This brood-guarding behavior reduces male activity ranges and increases their concealment, thereby decreasing their susceptibility to capture by fishing gear [46]. Meanwhile, females exhibit broader foraging movements to meet the high energetic demands of vitellogenesis, leading to their higher catch rates during the early reproductive period.

This nest-guarding behavior has been shown to enhance egg hatching success and maximize the reproductive investment of females during the breeding season. Gonadal developmental stages and gonadosomatic index (GSI) values revealed a unimodal annual reproductive cycle. From August onward, ovaries were predominantly at Stage II, accompanied by the lowest GSI values of the year. The proportion of Stage III ovaries gradually increased thereafter, with a concurrent gradual rise in GSI. The first appearance of Stage IV ovaries in January of the following year indicated the full initiation of vitellogenesis [47,48,49]. During the subsequent spring (March to June), the proportion of individuals at Stages IV–V increased rapidly, reaching a peak in May. This period coincided with the highest GSI values (exceeding 10%), reflecting final gonadal maturation and concentrated gamete release [50]. By July, most ovaries had regressed to Stage VI, accompanied by a sharp decline in GSI, marking the end of the annual reproductive cycle. Increasing day length in spring has been shown to elevate metabolic rates and stimulate gonadal endocrine activity, thereby supplying the energy and hormonal support necessary for final oocyte maturation [51,52]. Conversely, declining water temperatures in autumn suppress the pituitary–gonadal axis, leading to the cessation of reproductive activity [53,54]. The synchronous peak in GSI and the high prevalence of Stages IV–V gonads provide independent physiological validation of the breeding period identified by histological staging [55]. This alignment further underscores the spatiotemporal coordination between male nest-guarding behavior and female reproductive investment. Therefore, the current fishing moratorium from March to June, coupled with targeted protection of nest-guarding males aged two years and older, is expected to enhance female reproductive success and preserve the benefits of male parental care. These measures are likely to significantly support the sustainable recruitment of *O. potamophila* in Nansi Lake.

### 4.2. Cooperative Regulation of Sexual Maturity Threshold and Reproductive Output

In this study the initial sexual maturity body lengths for male and female *O. potamophila* in Nansi Lake were determined to be 73.59 mm and 73.66 mm, respectively, with a difference of less than 0.1 mm. It is widely recognized that body size, rather than age, serves as the primary indicator of sexual maturation in fishes [56]. Despite reaching maturity at a similar size threshold, the two sexes exhibit markedly divergent developmental trajectories. Below this size threshold, the proportion of mature females consistently exceeds that of males. Once the threshold is surpassed, however, males undergo rapid gonadal development, achieving maturation over a significantly shorter cycle compared to females (Figure 5). Histological observations reveal that females initiate maturation earlier, with a high prevalence of Stage III ovaries beginning in November, and some individuals retaining Stage VI features as late as July the following year. In contrast, males predominantly remain at Stage II from July to October, but accelerate rapidly into Stages III–IV after November (Figure 3). This “female-initiated, male-accelerated” maturation strategy minimizes inter-sexual timing differences in gonad development, thereby facilitating synchronized spawning and enhancing reproductive efficiency.

The absolute fecundity (AF) of *O. potamophila* in Nansi Lake ranged from 643 to 8904 eggs, with a mean value of 2306 ± 1430 eggs. Relative fecundity (RF) varied from 31.84 to 186.36 eggs/g, averaging 91.20 ± 40.09 eggs/g. Compared to the Taihu Lake population (RF = 87 eggs/g), the RF in Nansi Lake is slightly higher, yet it remains lower than that of the Jiangxi River system (RF = 121 eggs/g). Notably, the AF in Nansi Lake is the lowest among the three regions, suggesting that intraspecific variation in reproductive output is influenced by regional differences in prey availability and environmental conditions [23,24,25,26]. Furthermore, the AF of the wild population in Nansi Lake was significantly higher than that reported for eco-friendly aquaculture populations (2 years old: 2570 ± 943 eggs; 1 year old: 1157 ± 666 eggs) [57]. This disparity implies that dietary diversity and nutritional quality directly affect energy allocation to gonad development [58]. Natural selection factors such as predation pressure, disease prevalence, and resource competition may drive wild populations to adopt a strategy of high reproductive investment to compensate for adult mortality under environmental stress, thereby maintaining population replenishment over evolutionary time [59,60,61].

The age-dependent pattern reveals a clear energy allocation trade-off, characterized by an exponential increase in absolute fecundity with age: 1819 ± 839 eggs in 1-year-old individuals, 3176 ± 1319 eggs in 2-year-olds, and 6006 ± 4097 eggs in 3-year-olds. Linear modeling indicated a strong positive correlation between absolute fecundity and both body weight (*R*^2^ = 0.61) and body length (*R*^2^ = 0.50, *p* < 0.01), confirming that energy reserves are a primary factor driving increased egg production. Relative fecundity rose rapidly between ages 1 and 2, but stabilized and slightly declined thereafter, suggesting that older individuals prioritize metabolic maintenance over further increasing reproductive output per unit weight. This pattern aligns with the principle of “isometric growth–allometric reproduction” [62,63]. Therefore, implementing selective fishing regulations that protect mature female *O. potamophila* is expected to enhance overall reproductive efficiency and contribute to population stability in Nansi Lake.

### 4.3. Reproduction Parameter-Oriented Resource Management Pathways and Future Prospects

*O. potamophila* exhibits a reproductive strategy characterized by early maturation at a small body size and high reproductive output, with notable sexual dimorphism in developmental timing. Vitellogenesis in females commences prior to reaching 73.59 mm standard length (SL), and individuals aged two years or older account for more than 80% of the total egg production. Absolute fecundity increased exponentially with body weight, indicating a life history tactic that combines early maturation with sustained high reproductive investment in later life stages, a key adaptation for population sustainability. Ecologically, this strategy maximizes reproductive output while minimizing structural size costs [61]. Management efforts should therefore prioritize three key interventions: a seasonal fishing ban during critical reproductive periods, enforcement of a minimum catch size to protect immature individuals, and protection of feeding habitats to ensure adequate nutrition for broodstock.

The fishing ban should be implemented throughout the entire period from gonadal maturation to fry dispersal, specifically from March to June annually. This interval corresponds to the critical window during which the average daily water temperature in the lake increases from 18 °C to 26 °C. Concurrently, the current minimum catch size should be raised from 60 mm to 80 mm SL, which corresponds to the average body length of two-year-old females, thereby preserving highly reproductive individuals. Upgrading fishing gear to enforce this regulation is both practical and feasible [64]. In artificial breeding programs, the peak gonadosomatic index (GSI) in May can serve as a reference for scheduling spawning batches. Water temperatures of 22–26 °C are recommended for induction, and broodstock should receive enhanced nutrition, including feed with ≥45% protein and high levels of highly unsaturated fatty acids (HUFA), starting 60 days prior to the spawning period [49]. Looking forward, integrating reproductive parameters with environmental monitoring data to develop population dynamic models will enable annual resource forecasts and support precision quota management. Such an approach will provide scientific and managerial foundations for the sustainable utilization of *O. potamophila*. Moreover, advance prediction of annual catch potential and optimization of quota allocation can further quantify this species’ supply potential and economic value in the edible fish market.

Nevertheless, several uncertainties remain to be addressed. It is unclear how rising water temperatures may reduce the reproductive window, or how male nesting behavior responds to fishing pressure, and the impact of food availability on the spawning period of this species as a shallow-water benthic predator. Long-term data are needed to assess these impacts. Future work should develop integrated models combining climate, reproduction, food supply, and fishing factors to evaluate population resilience under different management strategies. Such approaches will improve resource recovery efforts for *O. potamophila*.

## 5. Conclusions

Based on a study of 894 *O. potamophila* from Nansi Lake (2017–2018), this research reveals key reproductive traits. The sex ratio averaged 1.06:1, but peaked at 2.14:1 (female:male) in April. Both sexes matured at one year and approximately 73.6 mm standard length. Spawning occurred from March to June, peaking in May when the gonadosomatic index reached 28.92%. Fecundity ranged from 643 to 8904 eggs and increased with size and age. Fish aged two years or older produced over 80% of all eggs, although relative fecundity stabilized after age two, indicating a shift toward growth rather than reproduction in older individuals. These findings support the “early maturation and high productivity” reproductive strategy of *O. potamophila*. The results provide a scientific basis for setting fishing closures, adjusting size limits, and improving broodstock management to aid conservation of this and similar benthic species. However, the model does not incorporate the potential negative effects of warming rates and extreme hydrological events on egg hatching rates. Future research should validate the robustness of management assumptions under global warming scenarios using long-term data.

## Figures and Tables

**Table 2:** Monthly ratio of female to male (F:M) for *Odontobutis potamophila* collected from Nansi Lake from August 2017 to July 2018.

Month		Number of Fish		Sex Ratio (F:M)	χ^2^-Tests
		Female	Male		
2017	August	14	19	0.74	0.381
	September	17	29	0.59	1.592
	October	28	18	1.56	1.100
	November	33	48	0.69	1.400
	December	42	38	1.11	0.100
2018	January	22	15	1.47	0.668
	March	88	74	1.19	0.606
	April	75	35	2.14	7.521 *
	May	58	78	0.74	1.479
	June	29	38	0.76	0.607
	July	27	16	1.69	1.430
Total		433	408	1.06	0.372

Note: * indicates significance (*p* < 0.05).

## Data Availability

The data presented in this study are available on request from the corresponding author.
